# Beyond the canonical: The role of post-transcriptional regulation in drug-target interaction prediction

**DOI:** 10.1371/journal.pcbi.1014440

**Published:** 2026-06-22

**Authors:** Md Istiaq Ansari, Khandakar Tanvir Ahmed, Debby D. Wang, Kirill Medvedev, Wei Zhang

**Affiliations:** 1 Department of Computer Science, University of Central Florida, Central Florida Blvd, Orlando, Florida, United States of America; 2 School of Science and Technology, Hong Kong Metropolitan University, Ho Man Tin, Hong Kong; Indonesia International Institute for Life Sciences, INDONESIA

## Abstract

Protein isoforms produced from the same gene through post-transcriptional regulatory mechanisms, such as alternative splicing, can substantially alter protein structure and function, including drug-binding properties. However, most existing drug-target interaction (DTI) and drug-target affinity (DTA) prediction models rely exclusively on a single representative protein sequence per gene, typically the canonical or longest isoform, thereby overlooking the functional diversity introduced by alternative isoforms. This assumption can introduce bias, limit generalizability, and compromise the biological validity of model predictions. In this study, we systematically investigate the impact of protein isoform variation on DTI prediction accuracy. Our results show that substituting the canonical sequence with an alternative isoform often leads to substantial declines in predictive performance. Structural and binding affinity analyses further reveal that these discrepancies are frequently associated with changes in predicted binding-site configurations, which we further examine through controlled perturbations of binding-site residues. These experiments suggest that even subtle alterations in binding regions can lead to inconsistent DTI predictions. Overall, our findings uncover a critical limitation in current DTI modeling frameworks and underscore the importance of incorporating isoform-specific information to better reflect biological reality and improve therapeutic relevance. The codes and datasets are available at https://github.com/compbiolabucf/DTIVariant.

## Author summary

Computational drug–target interaction models have become central tools in modern drug discovery, yet they typically represent each gene with a single canonical protein sequence, overlooking the fact that most human genes produce multiple isoforms through alternative splicing. These isoforms can differ in structure and drug-binding properties, raising the question of whether current prediction models are robust to such biologically realistic variation. Here, we systematically evaluate how replacing canonical sequences with alternative isoforms from the same gene affects both drug–target interaction and drug–target affinity predictions. We find that even when interaction labels are kept unchanged, isoform substitution often leads to substantial performance declines. Structural comparisons suggest that prediction consistency is closely linked to preservation of binding-site geometry, and controlled removal of binding-site residues progressively disrupts both interaction classification and affinity estimation. Together, our findings expose a fundamental limitation of gene-level modeling assumptions and highlight the need for isoform-aware datasets and prediction strategies to better reflect the functional complexity of the human proteome.

## Introduction

*In silico* drug-target interaction (DTI) and drug-target affinity (DTA) prediction have become a cornerstone of modern drug discovery, enabling rapid identification of therapeutic candidates and supporting drug repurposing at scale [1–3]. Over the past decade, substantial progress has been made in DTI modeling, leading to remarkable improvements in predictive accuracy. However, most existing models represent each gene using a single protein sequence, typically the canonical or longest isoform, while neglecting a fundamental aspect of molecular biology: alternative splicing [4]. This post-transcriptional mechanism generates multiple protein isoforms from the same gene, often with distinct structures and biological functions, including drug-binding properties [4,5]. As a result, the protein representation used by many DTI models may not reflect the actual isoform expressed in a given biological context.

Alternative splicing affects over 95% of human multi-exon genes [6], producing isoforms that can differ substantially from the canonical sequence in amino acid composition and domain architecture. Such changes may lead to structural rearrangements that modify existing binding sites or introduce novel ones, thereby altering ligand interactions. Importantly, isoform expression is context-dependent and varies across tissue types, developmental stages, and disease conditions [7,8]. For example, the isoTWAS framework [9] identified disease-associated isoforms, such as AKT3 and CUL3 in schizophrenia, that are undetectable at the gene level.

Although isoform-resolved DTI annotations remain limited, accumulating evidence suggests that isoform diversity can substantially influence protein function and drug response. ASpdb [10] provides structural resources for isoforms, including 3,400 canonical isoforms with experimental and predicted structures and more than 7,200 alternative isoforms with predicted structures generated using AlphaFold2 [11]. While ASpdb primarily supports the study of protein–protein interactions and does not include isoform-level DTI annotations [10], it reflects the growing availability of isoform structural information. Complementary studies have examined how alternative splicing reshapes protein structural features [12,13], revealing that isoform changes can disrupt functional regions relevant to molecular recognition. Venkat et al. [14] showed that alternative splicing of DCLK1 produces isoform-specific C-terminal tails that modulate kinase autoinhibition and enzymatic activity. Ha et al. [15] demonstrated that a splice variant of PIK3 CD (PI3Kδ-S) confers resistance to PI3Kδ inhibitors in African American prostate cancer patients through structural alterations in the binding domain. Similarly, Iqbal et al. [16] conducted a large-scale *in silico* screening of FDA-approved cervical cancer drugs and found that many compounds fail to effectively target all isoforms, particularly when alternative splicing disrupts canonical binding sites. Additional clinically validated examples illustrating how alternative splicing can alter drug binding, resistance, and target recognition are provided in Section A in S1 Text. Beyond biological observations, the necessity of isoform-level resolution is increasingly reflected in neighboring computational fields. Machine learning frameworks such as DeepISO [17] and DeepIII [18] have successfully modeled the ‘rewiring’ of protein-protein interactomes caused by alternative splicing, while functional annotation models like IsoGO [19] demonstrate that gene-level labels can be computationally decomposed into isoform-specific predictions. However, despite these advancements in protein function and interaction modeling, the impact of isoform variation remains largely unexplored in the specific context of DTI and DTA prediction.

However, this recognition has not yet been established in DTI and DTA prediction, where current models generally rely on a single protein sequence per gene obtained from reference databases such as UniProt [20]. Moreover, emerging DTI models that incorporate 3D structural features [21–23] are computationally expensive and are rarely applied in an isoform-resolved manner. To address these limitations, recent approaches increasingly leverage protein language models and large language models (LLMs), such as ESM-2 [24] and ChemBERTa [25], which are pretrained on large biomolecular corpora. These models can capture rich functional and structural signals directly from sequence and have shown promise in inferring aspects of 3D conformation. In principle, such models should be sensitive to isoform variation and enable isoform-specific DTI prediction. However, this assumption has not been systematically evaluated.

**Fig 1:**
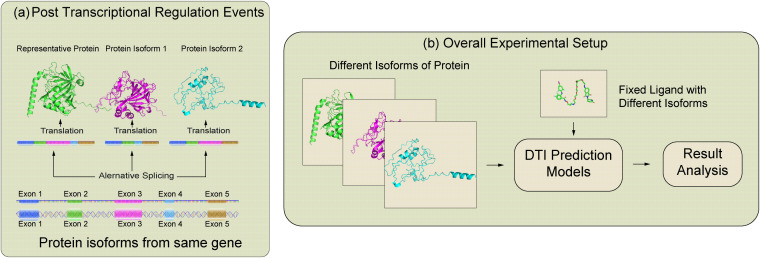
Overall pipeline of the experiments. **(a)** The figure shows how alternative splicing produces different isoforms from the same gene. **(b)** For a fixed ligand, we analyze how the DTI prediction model behaves for different isoforms of protein produced from the same gene as a result of post-transcriptional regulation events.

**Fig 2:**
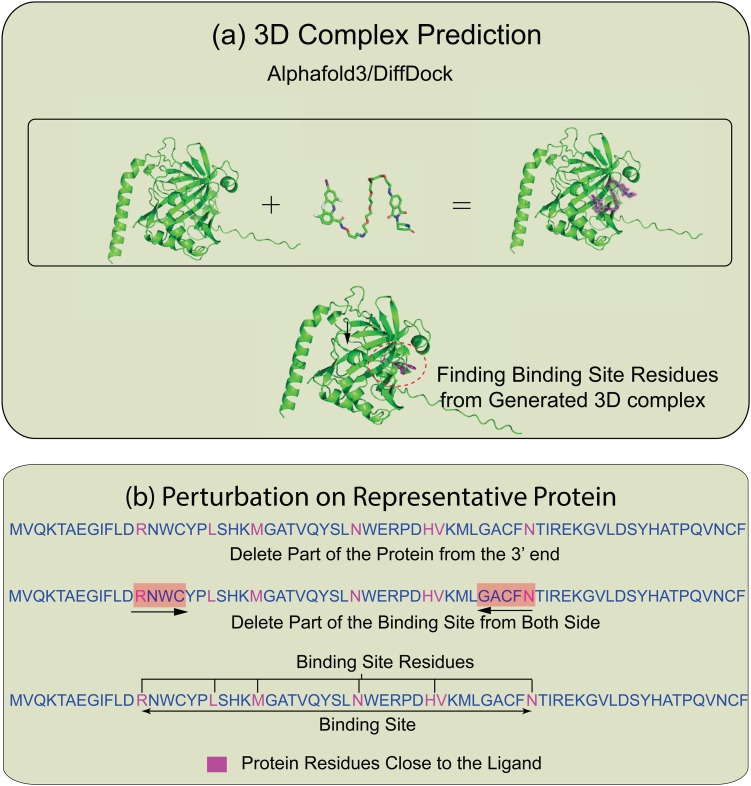
The figure shows the steps towards finding the binding-site residues for a given protein-ligand pair using computational methods. **(a)** The protein sequences are paired with ligands and fed to DiffDock/AlphaFold3 to generate the 3D complex with most probable binding pose. This 3D complex is used to calculate binding-site residues using a distance threshold from the ligand atoms. **(b)** The binding-site residues are used to modify the sequences with specific conditions relative to the binding site. Finally, representative protein, isoform protein, and modified protein sequences are fed through DTI prediction models to see how the results change.

In this study, we systematically investigate how protein isoform variation affects DTI prediction performance. We examine whether replacing canonical sequences with alternative isoforms leads to changes in prediction accuracy and identify structural and functional factors associated with these shifts (Fig 1). We further introduce controlled perturbations (Fig 2) in binding-site regions to assess model sensitivity to local changes in drug-interaction determinants. Through this comprehensive evaluation, we aim to reveal hidden biases in current DTI models and assess their robustness under biologically realistic isoform variation. Our findings underscore the need for isoform-aware modeling strategies that better capture the functional complexity of the human proteome and improve the precision of computational drug discovery.

## Results

### Protein isoform substitution reveals sensitivity in DTI models

Protein-coding genes frequently produce multiple protein isoforms through alternative splicing, many of which differ substantially in amino acid sequence, three-dimensional structure, and functional binding regions [26]. Despite this biological diversity, most DTI datasets and models implicitly assume that a single canonical protein sequence sufficiently represents all isoforms of a gene [1,27]. This assumption raises an important question: are DTI prediction models robust to isoform-level variation, or do they implicitly rely on sequence-specific binding patterns tied to a particular isoform? If alternative isoforms exhibit distinct binding behaviors, then substituting isoforms without updating interaction labels may expose vulnerabilities in current DTI modeling pipelines.

To investigate this question, we evaluated two representative DTI prediction models, DTI-LM [28] and MolTrans [29], on the BindingDB and DrugBank datasets. Our analysis focuses on how the model performance changes when canonical protein sequences are replaced with alternative isoforms, while interaction labels are kept unchanged. The overall pipeline is shown in Fig 1. Quantitative results are summarized in Tables 1 and 2.

**Table 1:** DTI Prediction Performance of DTI-LM and MolTrans for DrugBank dataset. Representative isoforms (Rep. iso) and Variant isoforms (Var. iso) are used in different combination for training and testing.

Model	Training Data	Test Data	TN	FP	FN	TP	F1	AUC
DTI-LM	Rep. iso	Rep. iso	635	86	79	687	0.893	0.945
	Rep. iso	Var. iso	528	72	381	266	0.540	0.712
	Var. iso	Rep. iso	580	141	194	572	0.773	0.856
MolTrans	Rep. iso	Rep. iso	603	102	131	636	0.845	0.908
	Rep. iso	Var. iso	569	30	508	141	0.344	0.647
	Var. iso	Rep. iso	600	102	296	474	0.700	0.814

True Negative (TN), False Positive (FP), False Negative (FN), True Positive (TP), F1 and AUC score are reported in the table.

**Table 2:** DTI Prediction Performance of DTI-LM and MolTrans for BindingDB dataset. Representative isoforms (Rep. iso) and Variant isoforms (Var. iso) are used in different combination for training and testing.

Model	Training Data	Test Data	TN	FP	FN	TP	F1	AUC
DTI-LM	Rep. iso	Rep. iso	439	69	52	463	0.884	0.953
	Rep. iso	Var. iso	391	35	214	274	0.688	0.864
	Var. iso	Rep. iso	431	77	112	403	0.810	0.908
MolTrans	Rep. iso	Rep. iso	418	52	38	276	0.860	0.945
	Rep. iso	Var. iso	353	41	130	148	0.634	0.833
	Var. iso	Rep. iso	415	55	75	239	0.786	0.908

True Negative (TN), False Positive (FP), False Negative (FN), True Positive (TP), F1 and AUC score are reported in the table.

As a baseline, we trained and evaluated both models using the default dataset configuration, in which each protein is represented by its canonical (representative) isoform (Rep. iso). Under this setting, DTI-LM achieves strong performance on DrugBank, with an AUC of 0.945 and an F1 score of 0.893, reflecting the standard evaluation assumption that representative isoforms adequately capture drug-target interactions. However, when the test set proteins are replaced with alternative isoforms (Var. iso) while keeping the training data and interaction labels unchanged, performance degrades sharply. In this setting, the F1 score drops to 0.540 and the AUC decreases to 0.712. This pronounced decline is primarily driven by a large increase in false negatives, indicating that the interactions when confronted with isoform variants has been changed and the unchanged labels no longer truly represents the interaction with the isoforms. These results suggest that isoform-specific sequence and structural differences can substantially alter binding behavior, thereby violating the assumption that interaction labels derived from canonical sequences generalize across isoforms.

To further probe the impact of isoform variability, we conducted an additional experiment in which a subset of representative protein sequences in the training data were replaced with variant isoforms, while the interaction labels were left unchanged. In this configuration, testing was performed on representative isoforms. For DTI-LM on DrugBank, this perturbation results in a reduction of the F1 score to 0.773 and the AUC to 0.856, which is notably worse than the baseline Rep. iso (train set) → Rep. iso (test set) setting. Because alternative isoforms may exhibit different binding affinities or contact regions, pairing them with labels derived from representative sequences effectively introduces noise into the training data. The observed performance degradation indicates that even partial isoform substitution during training can impair the model’s ability to learn consistent interaction patterns and generalize reliably. This explains why the Var. iso (train set) → Rep. iso (test set) setting exhibits a performance decline, although the decrease is less pronounced than in the Rep. iso (train set) → Var. iso (test set) setting. The asymmetry between these two scenarios arises from the extent of isoform replacement. When variant isoforms are introduced during training, only a subset of protein sequences is replaced—specifically, those for which variant sequences are available in the dataset—thereby maintaining partial alignment between the training and reference isoform distributions. In contrast, when variant isoforms are introduced in the test set, we ensured that every protein had at least one corresponding variant sequence, resulting in a more comprehensive distribution shift at evaluation time. Consequently, the performance drop is more substantial in the second row compared to the third row.

Importantly, the same qualitative trends are observed for MolTrans and across the BindingDB dataset. In all cases, replacing representative isoforms with variants either during training or testing leads to consistent reductions in F1 and AUC scores, as shown in Tables 1 and 2. Together, these results demonstrate that DTI models are highly sensitive to isoform-level perturbations and implicitly rely on isoform-specific sequence representations rather than gene-level abstractions. We have confirmed that the results with different settings are statistically significant by McNemar’s test. To further assess the robustness of the reported performance differences, we performed a bootstrap-based uncertainty analysis of the DTI results, and the details are provided in Section B in S1 Text.

Taken together, our findings indicate that alternative protein isoforms from the same gene do not necessarily share identical binding characteristics, and that ignoring isoform-level distinctions can introduce both evaluation bias and label noise in DTI prediction tasks. Additionally, to assess whether this effect could be explained by similarity between test proteins and proteins seen during training, we compared each test protein to its most similar training protein and analyzed the resulting similarity scores in relation to prediction and label agreement. As described in Section C in S1 Text, the similarity distributions across these categories overlap substantially, suggesting that high similarity to a training-set protein alone does not explain whether predictions remain stable or change after isoform substitution. These observations underscore the need for future DTI modeling pipelines to explicitly account for protein isoform diversity either through isoform-aware labeling strategies or sequence- and structure-sensitive modeling approaches to improve biological realism and predictive reliability.

### Preserved binding geometry underlies prediction consistency across isoforms

The isoform perturbation experiments reveal that DTI prediction models are sensitive to protein sequence variation, but they do not explain *why* certain isoform substitutions preserve predictions while others cause drastic changes. This raises a central mechanistic question: are consistent DTI predictions driven primarily by overall sequence similarity, or by the preservation of binding-site level structural features? To address this question, we perform a comparative structural analysis to link prediction consistency directly to three-dimensional protein–ligand geometry.

We first evaluate the model on a test set from BindingDB containing representative protein sequences and then replace each representative protein with its corresponding isoform variant, keeping the ligand unchanged. By comparing prediction outcomes from DTI-LM before and after isoform substitution, we identify cases where predictions remain stable and cases where they diverge. Based on this comparison, protein–ligand pairs are partitioned into two categories: *Matched Pairs*, for which the model produces identical predictions for representative and variant proteins, and *Mismatched Pairs*, for which the predictions differ. This categorization allows us to examine whether structural similarity at the binding interface explains prediction robustness.

**Fig 3:**
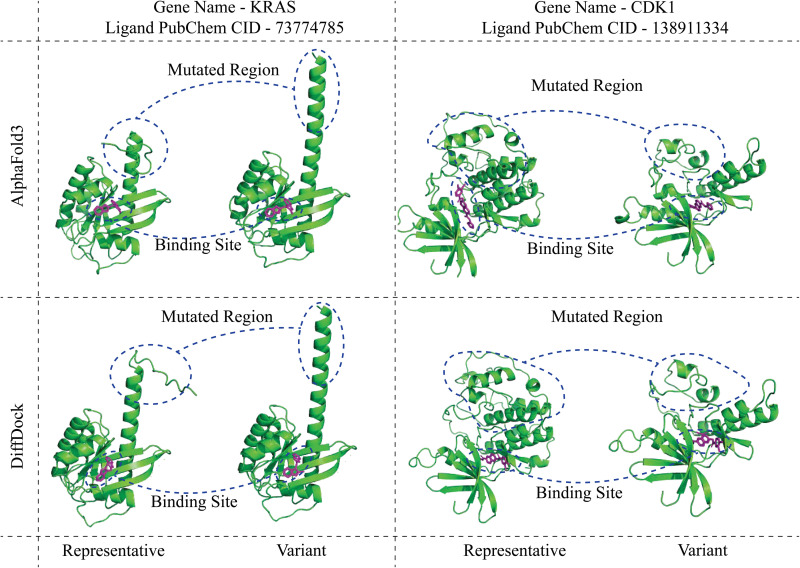
Structural comparison of protein–ligand complexes for Matched DTI prediction pairs, where the representative and the variant protein yield same prediction. Representative and variant protein pairs are shown in 3D in complex with a specific ligand. The 3D complexes are generated using both AlphaFold3 and DiffDock.

**Fig 4:**
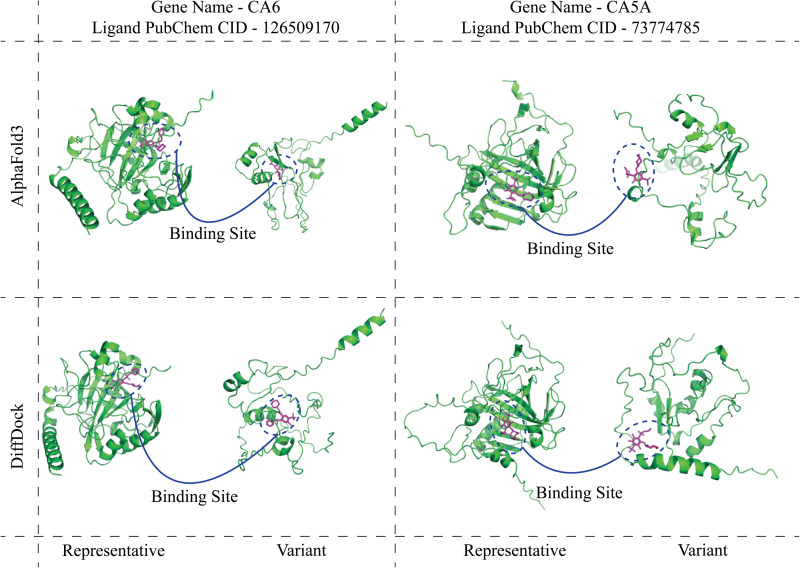
Structural comparison of protein–ligand complexes for Mismatched DTI prediction pairs, where the representative and the variant protein yield opposite prediction. Representative and variant protein pairs are shown in 3D in complex with a specific ligand. The 3D complexes are generated using both AlphaFold3 and DiffDock.

**Fig 5:**
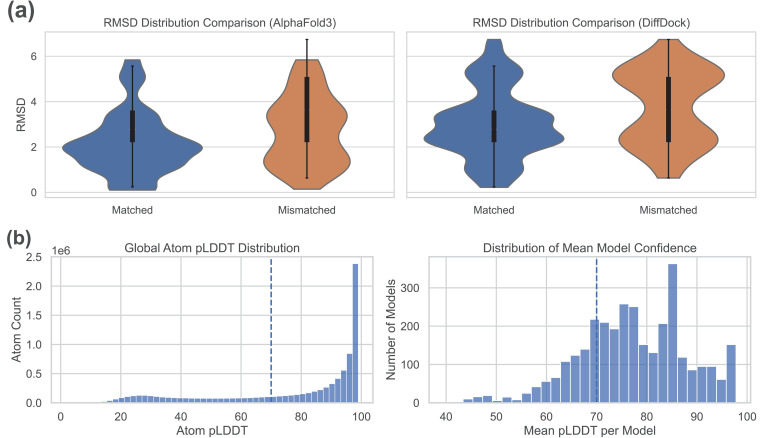
Figure (a) shows the RMSD distribution calculated between each representative vs variant protein structure pair interacting with same ligand in the test set. The 3D structures are predicted using AlphaFold3(left) and DiffDock(right) in combination with ESM2. The blue violin plot is for the pairs where representative and variant both yield same prediction and the orange violin plot is for the pairs where representative and variant yield different result. Figure (b) shows per atom pLDDT score (left) distribution and per model average pLDDT score (right) for the complexes predicted by AlphaFold3. There is a cutoff line shown at 70 for the confidence score.

For each representative–variant protein pair, three-dimensional protein–ligand complexes are generated using DiffDock [30] and AlphaFold3 [31]. These complexes are then aligned and visualized using PyMOL [32] to enable direct structural comparison. Representative examples from the *Matched Pairs* and *Mismatched Pairs* sets are shown in Fig 3 and Fig 4, respectively. Because alternative isoform structures may be more difficult to predict reliably than representative isoform structures, these structural comparisons are interpreted as qualitative and hypothesis-generating rather than definitive evidence of true isoform conformations. To show how confidently AlphaFold3 predicted these 3D complexes we show per atom pLDDT and per model average pLDDT distribution in Fig 5(b).

Across the *Matched Pairs* set, visual inspection reveals a high degree of structural conservation between representative proteins and their isoform variants, particularly in the vicinity of the ligand-binding site. Structural similarity distribution calculated from the 3D complexes is shown in Fig 5(a) for both AlphaFold3 and DiffDock. Both plots show a high density area at a higher RMSD for the mismatched pairs of representative-variant proteins interacting with the same ligand. Despite sequence-level differences introduced by alternative splicing, the overall binding-site geometry and residue-level spatial organization remain largely preserved. This structural consistency provides a plausible explanation for the stability of model predictions, suggesting that DTI models implicitly rely on conserved three-dimensional interaction patterns rather than exact sequence identity. RMSD distribution for the binding-site specific region is provided in Section D in S1 Text.

In contrast, protein pairs in the *Mismatched Pairs* sets exhibit pronounced structural divergence. These differences include reduced global alignment, partial loss of secondary structural elements, and substantial rearrangements or disruptions of the binding-site region. Such alterations are likely to modify the physicochemical environment experienced by the ligand, leading to changes in predicted interaction outcomes. The strong correspondence between structural divergence and prediction inconsistency suggests that binding-site architecture plays a central role in determining DTI model behavior. Some predicted variant isoform structures, such as the CA6 example in Fig 4, contain apparently dissociated secondary-structure elements. These cases may reflect genuine disruption caused by isoform sequence changes, but they may also arise from limitations of current structure prediction methods when applied to non-canonical isoforms. We therefore treat such examples as indicators of possible structural instability or modeling uncertainty rather than conclusive structural models.

Interestingly, we also observe a subset of *Matched Pairs* in which representative and variant proteins differ markedly in overall structure, yet still yield consistent predictions. In these cases, although the original binding site present in the representative protein may be altered or absent in the variant, alternative binding pockets or compensatory structural rearrangements appear to support similar ligand interactions. This observation highlights that structural divergence does not necessarily imply functional divergence, and suggests that DTI models may, in some cases, capture higher-level interaction features that transcend a single canonical binding site.

Taken together, this analysis suggests that prediction robustness in DTI models is more closely associated with the preservation of binding-site–level structural features than with sequence similarity alone. We additionally compared protein language model embeddings between representative and variant isoforms and found that most pairs remain close in embedding space, with high cosine similarity and low cosine distance, while a smaller subset shows larger embedding-level divergence (Section E in S1 Text). These findings provide a structural explanation for the isoform sensitivity observed in earlier experiments and emphasize the importance of incorporating three-dimensional context when evaluating model robustness to biologically realistic protein variation.

### Binding-site preservation supports reliable DTI predictions

While protein sequences encode global structural and functional information, DTIs are ultimately mediated by a relatively small subset of residues forming the ligand-binding site. This raises an important question for DTI modeling: to what extent do prediction models rely on binding-site–specific information, and how robust are they to partial disruption of this region? To address this, we conduct a controlled ablation study that systematically removes binding-site residues from protein sequences and evaluates how prediction accuracy degrades as critical interaction-related information is progressively lost.

Binding-site regions for each protein–ligand pair are identified using two complementary structure prediction tools, DiffDock [30] and AlphaFold3 [31], which generate plausible three-dimensional docking poses for a given protein–ligand pair. From the resulting protein–ligand complexes, binding-site residues are defined as all protein residues located within 5Å of any ligand atom. These residues collectively form a contiguous binding-site segment that serves as the target of subsequent perturbations.

The DTI model is trained exclusively on representative protein isoforms using unmodified sequences and ground-truth interaction labels. During evaluation, however, increasing fractions of the binding-site segment are removed from the protein sequence while keeping both the ligand and the interaction label unchanged. In the primary setting, binding-site residues are removed symmetrically from both ends of the identified segment, with deletion levels ranging from 0% to 100% in 10% increments. This design allows us to isolate the effect of progressively erasing binding-related information while minimizing confounding changes to the remainder of the protein sequence. The resulting performance trends are shown in Fig 7(a) and Fig (b).

As the proportion of removed binding-site residues increases, where binding sites are defined by DiffDock or AlphaFold3, model performance declines progressively. This decline is marked by a steady reduction in true positive predictions and a corresponding increase in false negatives. Removing binding-site residues eliminates interaction-relevant information, biasing the model toward the negative class, which becomes more likely in the absence of key binding features. Because the ground-truth labels for the variants remain identical to those of the canonical protein sequences, this shift in predictive bias leads to an elevated false-negative rate.

To further probe positional sensitivity within the binding site, we repeat the experiment using an asymmetric perturbation strategy in which residues are removed exclusively from the C-terminal end of the protein sequence. The resulting performance trajectory, shown in Fig 7(c), differs markedly from the symmetric deletion case. Model performance remains relatively stable up to approximately 40% deletion, after which a smooth and consistent decline is observed. This observation suggests that removing one end of the protein sequence does not impact binding until the deletion reaches the actual binding-site region.

This behavior suggests that residues near the C-terminal end of the binding-site segment contribute less critically to interaction prediction, allowing the model to tolerate substantial truncation before performance degrades. Beyond the 40% threshold, however, progressive removal of essential interaction-related residues leads to predictable loss of accuracy. The absence of a sharp transition indicates that, in this region, no single residue dominates prediction outcomes; instead, accurate DTI inference depends on the cumulative contribution of multiple binding-site residues.

Overall, these results indicate that DTI prediction models are highly sensitive to the integrity of the binding-site region, with a particularly strong dependence on a small core subset of residues. The asymmetric effects observed under different removal strategies further reveal that binding-site contributions are spatially heterogeneous. Together, these findings provide mechanistic insight into how sequence-level perturbations translate into prediction failures and highlight the importance of binding-site–aware modeling for robust and biologically grounded DTI prediction.

### Impact of progressive protein sequence deletion on affinity prediction

**Fig 6:**
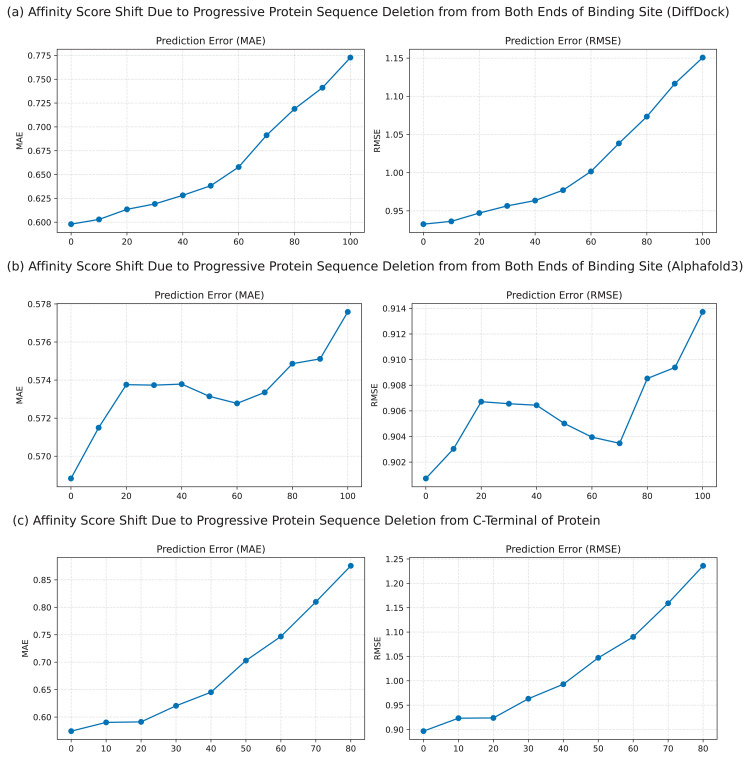
Effect of gradually removing parts of the protein predicted affinity values. AttentionDTA is used for prediction. MAE and RMSE values are shown against increasing percentage of length deletion with 10% increment on each step. In **(a)** and **(b)**, part of the protein is deleted from both sides of the binding-site region with increasing percentage, where the binding-site is calculated using **(a)** DiffDock and **(b)** AlphaFold3. **(c)** Protein segment is deleted with increasing length from the C-terminal end of the sequence.

Similar to the experiments described in section Binding-Site Preservation Supports Reliable DTI Predictions, we design another experiment where we measure how the drug-target affinity prediction changes with progressive deletion of protein sequence. The goal of this experiment is to assess the sensitivity of affinity prediction methods on progressive loss of binding-site residues from the protein sequence. We chose AttentionDTA [33], which models drug-target interactions by jointly encoding the ligand and protein sequences using attention-based neural architectures that learn context-aware representations of both modalities. The model applies cross-attention mechanisms to capture fine-grained interactions between ligand substructures and protein residues, enabling it to focus on the most relevant regions contributing to binding. These attended representations are then aggregated and passed through fully connected layers to predict the interaction affinity. We train and test using the same setup as described in section Binding-Site Preservation Supports Reliable DTI Predictions. The mean absolute error (MAE) and root mean squared error (RMSE), averaged over the entire test set, are reported to quantify the deviation between predicted and true affinity values under progressive sequence perturbation. The results are presented in Fig 6.

As seen from Fig 6(a) and (b), the affinity prediction error increases as the binding-site region is deleted progressively. Although DiffDock-based binding-site deletion results in a steady increase in MAE from 0.60 to 0.77, AlphaFold3-defined binding-site removal produces only negligible changes in performance. This discrepancy likely reflects differences in how the two methods delineate protein regions that are most informative for ligand interactions. DiffDock-derived binding sites may represent a more compact and interaction-focused subset of residues, such that their gradual removal leads to a proportional loss of predictive signal. In contrast, AlphaFold3-defined regions may encompass residues whose removal exerts a weaker immediate effect on the learned sequence–ligand representations. Consequently, affinity predictions remain comparatively robust to deletions based on AlphaFold3 annotations until more substantial portions of the sequence are perturbed.

As shown in Section Binding-Site Preservation Supports Reliable DTI Predictions, progressive deletion from the C-terminal end produces minimal changes in prediction accuracy at low deletion levels. However, once the truncation extends into the binding site region, the prediction error increases substantially, exceeding an MAE of 0.85 at higher deletion percentages (Fig 6(c)). These results indicate that affinity prediction is relatively robust to peripheral sequence truncation, but highly sensitive to deletions that disrupt binding site residues.

Overall, these observations suggest that affinity prediction performance is influenced more by perturbations affecting interaction-relevant regions than by sequence truncation at one end of the protein. Deletions that leave the binding-site region largely intact result in relatively stable predictions, whereas removal of residues overlapping the binding site leads to a measurable increase in prediction error. This indicates that sequence-based affinity models are particularly sensitive to localized disruptions of binding-related information, while exhibiting greater robustness to deletions outside these regions. Additionally, the differing trends observed under DiffDock- and AlphaFold3-derived binding-site definitions motivate further investigation into how these approaches characterize binding regions and how such differences influence downstream predictive modeling.

### Comparative analysis of binding-site sequence similarity

To analyze the differences of the protein sequences between the representative isoform and the variant isoform, we run sequence alignment. Pair-wise BLASTP alignment scores were calculated between the representative and variant isoforms. This was done separately for *Matched Pairs* and *Mismatched Pairs* set. The results are shown in Fig 8. The blue violin plot shows that values are concentrated at the higher end of the similarity range, meaning that the representative and variant protein pairs that produce the same interaction prediction with the same ligand are very similar, although some pairs have low similarity. This can again be explained similarly as we discussed before in section Preserved Binding Geometry Underlies Prediction Consistency Across Isoforms, that those variant proteins with very different structure or low sequence similarity with the representative might have gained a new or different binding-site.

The orange violin plot shows the similarity score for the mismatched pairs. The similarity score is distributed almost evenly all over the data space. This shows a less consistent similarity distribution between the representative and variant protein sequences for the pairs that produce different interaction prediction for representative and variant isoform.

## Discussion

This study sheds light on a fundamental issue in current DTI prediction pipelines. The common assumption that a canonical protein sequence can adequately represent all functional isoforms may need to be reconsidered. Our results show that different isoforms generated from the same gene are interpreted differently by DTI prediction models such as DTI-LM and MolTrans, leading to performance degradation when canonical sequences in the test set are replaced with isoforms.

First, we demonstrate that isoforms are handled differently by DTI models. When the representative protein sequence is replaced with an isoform sequence from the same gene, while keeping the interaction label unchanged, the model often produces a different prediction. In reality, different isoforms from the same gene may indeed interact differently with the same drug, and such differences ultimately require experimental validation. We further show that both isoform substitutions and small perturbations to the representative sequence can alter DTI predictions. Through controlled experiments, we evaluate the extent to which these perturbations influence model outputs. This observation echoes recent work on cryptic binding pockets [16] and highlights the need for DTI models to better account for structural flexibility and dynamic plasticity.

Since the binding-site information is not available in any public DTI datasets, we use DiffDock and AlphaFold3 to predict the 3D complex with best possible ligand pose. Traditional docking methods such as AutoDock Vina [34] rely on search algorithms and hand-crafted scoring functions to identify optimal ligand poses and they are often slow and struggle with accuracy due to the rugged energy landscapes and high-dimensional search space. It requires a manual definition of the binding pocket, a predefined search space, before the estimation. Recent deep learning methods that treat docking as a regression problem offer faster predictions but still fall short in accuracy and often yield physically implausible results due to the inability to capture uncertainty and multimodality in pose distributions. GNINA [35] is one of them, but it requires a predefined binding pocket to accurately generate the binding pose. DiffDock addresses these limitations by treating molecular docking as a generative modeling problem that learns a distribution over ligand poses conditioned on the protein structure, and AlphaFold3 similarly employs a diffusion-based framework to directly predict protein–ligand complex structures without the need for predefined binding pockets. We also explored AutoDock Vina [34] for this task but it showed poor performance and inconsistency along with the limitation of having to define the binding pocket, a predefined search space, manually before the estimation. DiffDock and AlphaFold3 are very consistent, accurate and convenient in predicting these 3D complexes [36,37].

However, we acknowledge that structure prediction for non-canonical and alternatively spliced protein isoforms remains an important limitation. Current structure prediction methods are generally optimized and benchmarked primarily on canonical or well-characterized protein sequences, and their reliability may decrease for alternative isoforms, particularly when splicing disrupts structured domains or removes stabilizing sequence context. Recent work [38] showed that protein language model–based structure predictors can predict alternative isoforms as folded fragments of their corresponding full-length proteins, even when such structures expose hydrophobic or aggregation-prone regions and may be physically implausible. This suggests that current models may rely, at least in part, on evolutionary statistics or segment-pairing patterns learned from full-length protein families rather than fully modeling the biophysics of isolated isoform folding. Therefore, the predicted isoform structures used in this study should be interpreted cautiously, especially in cases where variant structures appear fragmented or contain dissociated secondary-structure elements.

Structural comparison of *matched* and *mismatched* isoform-ligand pairs using AlphaFold3, DiffDock and PyMOL provides suggestive evidence that structural divergence may contribute to the observed prediction discrepancies, while recognizing that predicted isoform structures may contain modeling uncertainty. This is further supported by sequence similarity analysis of binding-site regions, where *mismatched* predictions correspond to lower BLASTP similarity scores. Although, there are some data points among the *matched* predictions with low similarity score, this can be caused by an emergence of a new binding-site in the isoform although the sequence has changed. This introduces additional complexity in the DTI research when dealing with isoforms.

The goal of this study is to draw attention to the sparse annotation of isoform-specific interactions in public databases, which limits the scope of supervised training for DTI models. One important factor that deserves more attention is that isoform expression patterns are often tissue- or disease-context dependent [7,8]. Developing context-aware interaction databases that explicitly annotate whether drug–target interactions are isoform-specific, and under what biological conditions they occur, would significantly benefit the field. Future datasets could include multiple isoforms from the same gene along with their interaction labels and binding-affinity measurements. Such resources would help clarify how post-transcriptional regulation alters protein function and drug interactions.

Although our structural and perturbation analyses support the computational relevance of isoform variation, the present study does not include experimental validation of isoform-specific drug–target interactions. Therefore, our findings should be interpreted primarily as evidence of model sensitivity under isoform substitution, and experimental confirmation of specific isoform–ligand interactions remains an important direction for future work. Future work should combine isoform-aware computational modeling with experimentally resolved isoform-specific interaction and affinity measurements to determine which predicted effects correspond to biologically realized interactions.

## Materials and methods

### Datasets

In this work, we use two publicly available datasets: DrugBank [39] and BindingDB [40]. BindingDB contains experimentally measured binding affinities between drugs and protein targets. On the other hand, DrugBank provides binary annotation for interaction. Both datasets provide protein-ligand pair with annotation label for each entry. After filtering out entries with missing values, invalid protein and SMILES sequences and extremely large affinity values, we chose proteins with a maximum length of 700 amino acids due to computational limitations. We also dropped entries with protein length less than 50 as they are considered peptides [41,42]. We apply these filters to both BindingDB and DrugBank datasets. From raw BindingDB 202406 data we get a dataset comprising 1,254 unique protein sequences interacting with 7,165 unique drugs and 32,601 interactions. Similarly, from DrugBank we get binary drug–target interaction annotations including 2,162 unique protein sequences interacting with 1,597 unique drugs with 12,083 interactions. For BindingDB, binding affinities are converted into binary interaction labels using a predefined threshold. Specifically, the dissociation constant (Kd) is used as the affinity measure, and interactions with Kd values below 100 nM are labeled as positive interactions, while the remaining pairs are treated as negative. In contrast, DrugBank provides binary interaction annotations by default and therefore does not require affinity thresholding.

We construct train–test splits such that all proteins in the test set have at least one variant protein along with the representative protein sequence from the same gene. Under this setting, the BindingDB dataset yields 18,192 training pairs and 1,000 testing pairs, while the DrugBank dataset yields 9,548 training pairs and 1,487 testing pairs.

We used the Ensembl database [43] to collect protein isoforms corresponding to each representative (canonical) protein encoded by the same gene. Ensembl is a comprehensive, open-access genome resource developed by EMBL-EBI that provides annotated genomic information, including transcript, protein, variant, and regulatory element data. It also incorporates annotations from the GENCODE project [44], which offers a detailed catalog of human and mouse gene annotations, including extensive splice variant information. In this study, protein sequences from BindingDB and DrugBank were treated as canonical sequences because they are associated with experimentally validated drug–target interaction labels. For each canonical protein, we identified the corresponding gene ID in Ensembl and retrieved all Ensembl protein sequences sharing that gene ID but differing from the canonical sequence; these were considered variant isoforms of the same gene. We included only canonical proteins with at least one mapped variant isoform in our experiments. To ensure meaningful comparisons, each variant isoform was required to be at least half the length of its corresponding canonical sequence.

### DTI prediction methods

For our experiments, we select two representative DTI prediction models as baselines: DTI-LM [28] and MolTrans [29]. DTI-LM is a language model–based DTI framework that employs ESM2 [24] as the protein encoder and ChemBERTa [25] as the molecular encoder. These encoders are pretrained on large-scale corpora, comprising approximately 138 million protein sequences for ESM2 and 77 million unique molecules in SMILES format for ChemBERTa. Owing to their extensive pretraining, both models provide rich contextual representations that are effective for downstream prediction tasks, even in low-data regimes. In DTI-LM, the protein and drug embeddings generated by these language models are subsequently processed by a graph attention module, and the final interaction affinity is predicted via a fully connected layer.

MolTrans similarly leverages large-scale unlabeled data, but focuses on learning a task-specific tokenizer that captures chemically meaningful substructures potentially relevant to drug-target interactions. These substructure representations are passed through a transformer-based architecture for feature extraction, followed by a prediction layer. By incorporating domain knowledge and large biomedical datasets, MolTrans aims to enhance both predictive performance and model interpretability.

**Fig 7:**
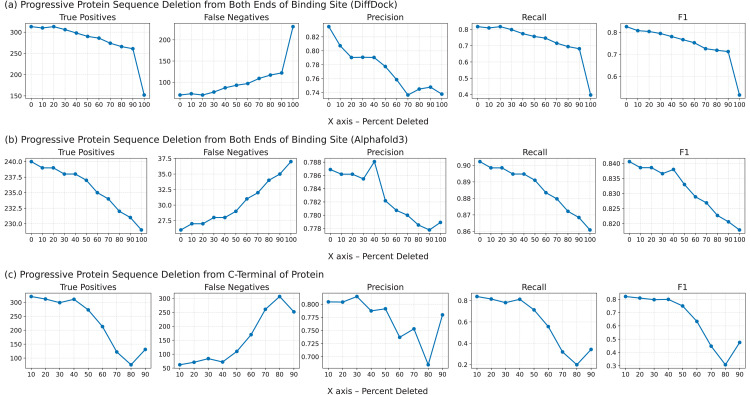
Effect of gradually removing parts of the protein on performance. The performance metrics are displayed against increasing percentage of length deletion with 10% increment on each step. In **(a)** and **(b)**, part of the protein is deleted from both sides of the binding-site region with increasing percentage, where the binding-site is calculated using **(a)** DiffDock and **(b)** AlphaFold3. **(c)** Protein segment is deleted with increasing length from the C-terminal of the sequence.

For evaluation, we employ standard metrics commonly used in DTI studies, including AUC and F1 score. In addition, we report the exact counts of the confusion matrix in Tables 1 and 2, and visualize the corresponding prediction shifts in Fig 7. This allows for a more detailed analysis of how prediction outcomes change when protein sequences are systematically modified, while the ground-truth interaction labels remain unchanged.

### Ligand docking and pose estimation with DiffDock and AlphaFold3

To visualize why DTI prediction models behave differently to the representative and the variant isoform we generate their 3D structures and inspect the binding-site differences when interacting with the same ligand. To generate three-dimensional protein–ligand complexes with plausible binding geometries at scale, we employ two complementary structure prediction approaches: DiffDock [30] and AlphaFold3 [31]. DiffDock is a diffusion-based generative docking framework that predicts ligand binding poses conditioned on protein structures by explicitly modeling ligand translation, rotation, and torsional degrees of freedom. However, DiffDock is not designed to generate 3D structures of a protein or a ligand from their sequence, it only provides a 3D pose for a given the 3D structures of protein-ligand pair. The pipeline provided by the authors uses ESM [24] to generate 3D structure of a protein from the sequence and RDKit [45] to generate 3D structure from a SMILES sequence. Starting from a randomized initial configuration, DiffDock iteratively refines ligand poses through a learned reverse diffusion process. It has demonstrated strong docking performance on the PDBBind benchmark, achieving a reported top-1 success rate of approximately 38% under the RMSD <2Å criterion [30].

In addition to DiffDock, we utilize AlphaFold3 [31], a unified diffusion-based model designed to predict the structures of biomolecular complexes, including protein–ligand assemblies. Unlike traditional docking approaches, AlphaFold3 directly predicts the full protein–ligand complex without requiring prior specification of a binding pocket. It has been shown to achieve near-experimental accuracy across a broad range of protein–ligand structure prediction tasks [31]. Employing both DiffDock and AlphaFold3 enables us to evaluate the consistency of inferred binding regions across distinct structure generation paradigms.

For each protein–ligand pair, we generate three-dimensional complexes using both DiffDock and AlphaFold3 and subsequently identify binding-site residues based on spatial proximity. Specifically, protein residues with any atom located within 5Å of a ligand atom are defined as binding-site residues as convention [46–48]. The binding region is represented as the contiguous sequence segment spanning these contact residues and is used in downstream structural analyses and controlled sequence perturbation experiments.

### DTI and DTA prediction under progressive binding-site deletion

We design this experiment to examine how perturbations to protein binding-sites affect DTI and DTA performance. Given a protein–ligand complex, we first identify binding-site residues and subsequently modify the corresponding protein sequence to investigate how DTI and DTA prediction models respond to controlled sequence perturbations. Using these binding-site annotations, we design two complementary experimental settings that progressively remove binding-related information from the protein sequence while keeping the ligand and interaction label unchanged. The process is shown in Fig 2.

In the first setting, we directly perturb the binding site by symmetrically removing residues from both termini of the binding-site region determined by DiffDock and AlphaFold3 (Fig 7(a) and Fig (b)). Specifically, if a protein consists of 100 residues and the predicted binding site spans residues 31–80, a 20% deletion corresponds to removing 10 residues in total out of the 50, 5 from the N-terminal side of the binding site (residues 31–35) and 5 residues from the C-terminal side (residues 76–80). This strategy enables a systematic evaluation of how prediction performance changes as core binding-site region is progressively deleted.

In the second setting, we examine the effect of global sequence truncation by removing an increasing fraction of residues from the C-terminal end of the protein sequence (Fig 7(c)). Unlike the binding-site–focused perturbation, this approach does not explicitly target contact residues, but instead probes the model’s sensitivity to distal sequence context and overall structural integrity, which may indirectly influence binding-site formation and recognition.

For each deletion level, the perturbed protein sequence and the original ligand are provided as input to DTI-LM for DTI prediction, which outputs a binary interaction label, and to AttentionDTA for DTA prediction, which outputs a continuous binding-affinity value. DTI performance is evaluated using true positives, false negatives, precision, recall, and F1 score. DTA performance is evaluated using mean absolute error (MAE) and root mean squared error (RMSE), computed between predicted and ground-truth affinities and averaged over the test set. By comparing error trends across binding-site–focused deletions and global truncations, as well as across DiffDock- and AlphaFold3-derived binding-site definitions, these experiments quantify the extent to which sequence-based DTI and DTA predictions depend on the preservation of binding-site residues.

**Fig 8:**
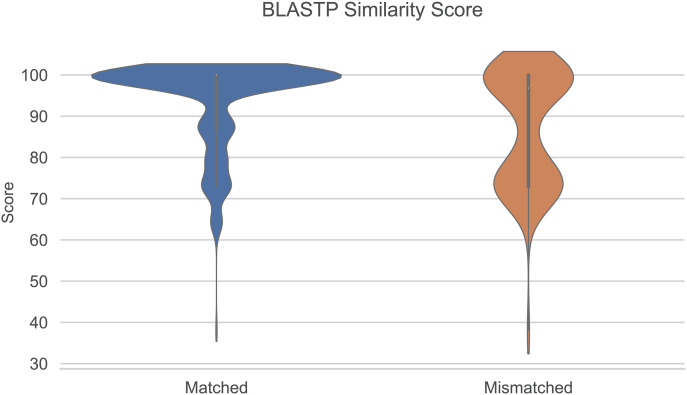
Violin Plot showing the distribution of BLASTP similarity score between the representative and variant proteins. The blue one is for the matched pairs and the orange plot is for the mismatched pairs.

To quantify sequence-level differences between representative and variant isoforms at the binding-site level, we compute pairwise similarity scores restricted to binding-site regions. For each protein–ligand pair, we extract the binding-site subsequence from both the representative and variant proteins using the corresponding three-dimensional complexes generated by DiffDock and AlphaFold3. The binding-site subsequence is defined as the contiguous amino acid segment spanning from the first to the last predicted binding-site residue. Pairwise sequence similarity is then assessed using BLASTP via the BioPython [49] NcbiblastpCommandline interface with default parameters. The resulting similarity distributions for matched and mismatched prediction pairs are summarized in Fig 8.

## Supporting information

S1 Text This supporting Document contains all supplementary analyses cited in the main text. It includes the following sections: Section A. Clinical evidence for the biological relevance of isoform-aware DTI prediction. Section B. Bootstrap-based uncertainty analysis of DTI performance. Section C. Effect of train-test sequence similarity. Section D. Global and binding-site specific RMSD. Section E. Protein language model embedding similarity between representative and variant isoforms.
(PDF)
