# Supplementary material for: Beyond the canonical: The role of post-transcriptional regulation in drug-target interaction prediction
Source: PLoS Comput Biol. 2026 Jun 22;22(6):e1014440. doi: 10.1371/journal.pcbi.1014440 (PMC13298989; doi:10.1371/journal.pcbi.1014440)
Supplement: S1 Text — It includes the following sections: Section A. Clinical evidence for the biological relevance of isoform-aware DTI prediction. Section B. Bootstrap-based uncertainty analysis of DTI performance. Section C. Effect of train-test sequence similarity. Section D. Global and binding-site specific RMSD. Section E. Protein language model embedding similarity between representative and variant isoforms. (PDF) [file pcbi.1014440.s001.pdf]

Supplementary Material for “Beyond the Canonical: The Role of Post-transcriptional Regulation in Drug-Target Interaction Prediction”

Md Istiaq Ansari, Khandakar Tanvir Ahmed, Debby D. Wang, Kirill Medvedev, Wei Zhang

June 15, 2026

A Clinical Evidence for the Biological Relevance of Isoform-Aware DTI Prediction

Alternative splicing can alter drug–target interactions by changing the sequence, structure, or availability of the molecular interface through which a therapeutic agent engages its target. These effects are not limited to minor quantitative changes in affinity. In clinically relevant settings, isoform variation can remove binding pockets, remodel ligand-specific recognition surfaces, alter regulatory domains that determine drug response, or delete epitopes required for antibody and cell-based therapies. These examples provide direct biological motivation for evaluating DTI models at the isoform level rather than assuming that a single canonical protein sequence adequately represents all targetable gene products.

1. **Complete loss of binding pockets: AR-V7 and enzalutamide.**

   A prominent example is the androgen receptor splice variant AR-V7 in castration-resistant prostate cancer. The canonical androgen receptor contains a C-terminal ligand-binding domain that is targeted by second-generation anti-androgens such as enzalutamide. In contrast, AR-V7 is generated by alternative splicing and lacks this ligand-binding domain. As a result, the physical binding pocket required for enzalutamide engagement is absent, while the receptor remains constitutively active through retention of its N-terminal transactivation and DNA-binding domains. In this setting, a DTI model that represents the target using only the canonical androgen receptor sequence may incorrectly infer drug sensitivity, even though the dominant disease-driving isoform is structurally incapable of binding the drug [1].

2. **Structural reconfiguration of binding specificity: FGFR2b/c.**

   Isoform-specific changes can also remodel binding specificity without eliminating the target protein. The *FGFR2* gene produces the FGFR2b and FGFR2c isoforms through mutually exclusive splicing of exons encoding part of the third immunoglobulin-like domain. This region contributes directly to ligand recognition, and the exon swap changes residues that shape the local binding interface. Consequently, FGFR2b and FGFR2c display distinct ligand-binding preferences despite sharing high overall sequence similarity. This example illustrates why global sequence similarity alone may be insufficient for DTI

prediction: a small isoform-specific change concentrated at the binding interface can substantially alter ligand compatibility and therapeutic targetability [2].

3. **Transition from inhibition to activation: BRAF V600E.**

   Alternative splicing can further change the functional consequence of drug engagement. In melanoma, resistance to vemurafenib has been linked to truncated BRAF V600E splice variants that lack regulatory regions required for normal control of dimerization. These shortened isoforms can form constitutively active dimers, reducing sensitivity to inhibitors designed to target the monomeric form of mutant BRAF. In this case, the relevant isoform does not merely weaken a predicted interaction; it changes the biological response associated with inhibitor exposure and contributes to therapeutic resistance [3]. This highlights an important limitation of gene-level target representations, because the same nominal target may exhibit different pharmacological behavior depending on the expressed isoform.

4. **Antigen escape via epitope deletion: CD19-Δex2.**

   Isoform-level effects are also relevant for biologic and cell-based therapies. In B-cell malignancies, alternative splicing of *CD19* can produce isoforms lacking exon 2, including CD19-Δex2. Although these variants may remain expressed on the cell surface, deletion of the exon can remove or alter the epitope recognized by CD19-directed chimeric antigen receptor T cells. The therapeutic interaction therefore fails not because the target gene is absent, but because the isoform-specific binding interface required for recognition has been modified [4]. This example reinforces the need for DTI pipelines to represent the actual protein sequence and binding interface present in the disease context.

B Bootstrap-Based Uncertainty Analysis of DTI Performance

To quantify uncertainty in the reported performance metrics, we performed bootstrap resampling of the test set and computed standard deviations and 95% confidence intervals for F1 and AUC. For the representative-to-representative setting, we obtained F1 $= 0.8254 \pm 0.0155$ with a 95% confidence interval of $[0.7954, 0.8549]$, and AUC $= 0.8581 \pm 0.0120$ with a 95% confidence interval of $[0.8356, 0.8813]$. For the representative-to-variant setting, we obtained F1 $= 0.7818 \pm 0.0161$ with a 95% confidence interval of $[0.7504, 0.8125]$, and AUC $= 0.8230 \pm 0.0123$ with a 95% confidence interval of $[0.7988, 0.8486]$.

These results indicate that the performance estimates are reasonably stable under resampling and that the decrease in performance after isoform substitution remains consistent under bootstrap evaluation. In addition, a paired statistical test on the binary predictions showed that the difference between the two settings is statistically significant.

C Effect of Train-Test Sequence Similarity

To assess whether sequence similarity between training and test proteins influences the observed effect of isoform substitution, we compared each test protein with the most similar protein in the training set. We then stratified the resulting maximum sequence similarity scores according to the agreement among three binary quantities: the test-set label, the test-set prediction, and the label of the most similar training protein.

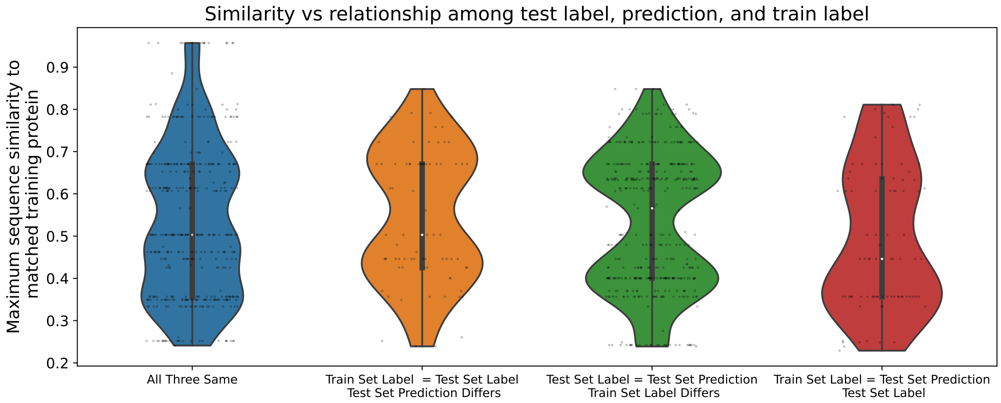

Fig A in S1 Text. Maximum sequence similarity between each test protein and its most similar training protein. Similarity scores are stratified by the relationship among the test-set label, test-set prediction, and label of the most similar training protein. The overlapping distributions indicate that train–test sequence similarity alone does not explain the prediction changes observed after isoform substitution.

As shown in Fig A in S1 Text, the similarity distributions across these categories overlap substantially. Cases in which the test label, test prediction, and matched training label all agree span a broad range of sequence similarity values, as do cases in which the test prediction differs despite agreement between the test label and matched training label. Similarly, categories involving disagreement between the test label and the matched training label also show overlapping similarity distributions. These results suggest that high similarity to a training-set protein does not, by itself, explain whether predictions remain stable or change after replacing representative isoforms with alternative isoforms.

D Global and binding-site specific RMSD

In this experiment we further analyzed binding-site-specific structural differences. In the main manuscript we report the overall RMSD distribution between the representative and variant protein for matched and mismatched groups. In this experiment we only focus on the binding-site specific RMSD distribution for the same experimental setup. The RMSD distribution shown in Fig B in S1 Text follows the same overall trend where the matched group shows a dense area towards lower RMSD region and the mismatched group has a peak towards the high RMSD region as well.

E Protein Language Model Embedding Similarity Between Representative and Variant Isoforms

To assess whether representative and variant isoforms occupy distinct regions of protein language model embedding space, we compared their embedding-level similarity using cosine similarity and cosine distance. For each representative–variant isoform pair, protein sequence embeddings were generated using a protein language model, and pairwise cosine similarity was calculated between the representative isoform embedding and its corresponding variant isoform embedding.

As shown in Fig C in S1 Text, most representative–variant isoform pairs exhibit high cosine similarity, with values concentrated near 1.0. Consistent with this observation,

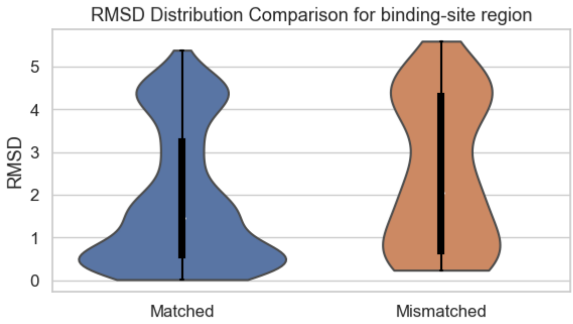

Fig B in S1 Text. Figure shows the RMSD distribution calculated between each representative vs variant protein's binding-site region when interacting with same ligand. The original manuscript reports the RMSD similarity for the whole protein while this plot shows the binding-site specific RMSD distribution.

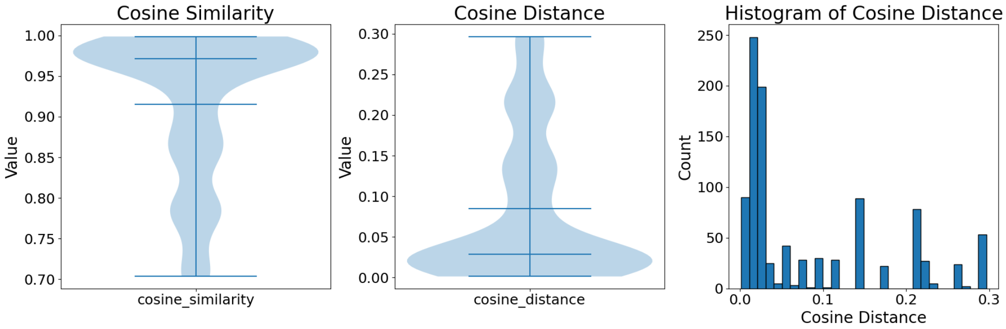

Fig C in S1 Text. Protein language model embedding similarity between representative and variant isoforms. The left panel shows the distribution of cosine similarity between representative–variant isoform pairs, the middle panel shows the corresponding cosine distance distribution, and the right panel shows the histogram of cosine distances. Most isoform pairs show high cosine similarity and low cosine distance, indicating close embedding-level correspondence, while a smaller subset shows greater divergence.

the corresponding cosine distances are generally low, with most pairs concentrated near 0. The histogram of cosine distances further indicates that the majority of isoform pairs fall within a low-distance range, although a smaller subset extends toward larger distances. These results suggest that representative and variant isoforms are often close in protein language model embedding space, while a subset of variants exhibit more substantial embedding-level divergence.

References

1. Antonarakis ES, Lu C, Wang H, Luber B, Nakazawa M, Roeser JC, et al. AR-V7 and resistance to enzalutamide and abiraterone in prostate cancer. New England Journal of Medicine. 2014;371(11):1028–1039. doi:10.1056/NEJMoa1315815.
2. Mohammadi M, Olsen SK, Ibrahimi OA. Structural basis for fibroblast growth factor receptor activation. Cytokine & growth factor reviews. 2005;16(2):107–137.
3. Poulikakos PI, Persaud Y, Janakiraman M, Kong X, Ng C, Moriceau G, et al. RAF inhibitor resistance is mediated by dimerization of aberrantly spliced BRAF (V600E). Nature. 2011;480(7377):387–390.
4. Sotillo E, Barrett DM, Black KL, Bagashev A, Oldridge D, Wu G, et al. Convergence of acquired mutations and alternative splicing of CD19 enables resistance to CART-19 immunotherapy. Cancer discovery. 2015;5(12):1282–1295.

Contents

A Clinical Evidence for the Biological Relevance of Isoform-Aware DTI Prediction 1

B Bootstrap-Based Uncertainty Analysis of DTI Performance 2

C Effect of Train-Test Sequence Similarity 2

D Global and binding-site specific RMSD 3

E Protein Language Model Embedding Similarity Between Representative and Variant Isoforms 3
